# Sub-Inhibitory Antibiotic Exposure and Virulence in *Pseudomonas aeruginosa*

**DOI:** 10.3390/antibiotics10111393

**Published:** 2021-11-13

**Authors:** Charlotte Nolan, Volker Behrends

**Affiliations:** School of Life and Health Sciences, University of Roehampton, London SW15 4JD, UK; nolanc@roehampton.ac.uk

**Keywords:** *P. aeruginosa*, antibiotics, cystic fibrosis, metabolism, virulence factors, azithromycin, context-dependence, sub-MIC, sub-lethal

## Abstract

*Pseudomonas aeruginosa* is a prime opportunistic pathogen, one of the most important causes of hospital-acquired infections and the major cause of morbidity and mortality in cystic fibrosis lung infections. One reason for the bacterium’s pathogenic success is the large array of virulence factors that it can employ. Another is its high degree of intrinsic and acquired resistance to antibiotics. In this review, we first summarise the current knowledge about the regulation of virulence factor expression and production. We then look at the impact of sub-MIC antibiotic exposure and find that the virulence–antibiotic interaction for *P. aeruginosa* is antibiotic-specific, multifaceted, and complex. Most studies undertaken to date have been *in vitro* assays in batch culture systems, involving short-term (<24 h) antibiotic exposure. Therefore, we discuss the importance of long-term, *in vivo-*mimicking models for future work, particularly highlighting the need to account for bacterial physiology, which by extension governs both virulence factor expression and antibiotic tolerance/resistance.

## 1. Introduction

Gram-negative bacteria pose a formidable clinical challenge, due to both intrinsic as well as acquired antibiotic resistance [1,2,3,4]. Hospital-acquired infections by opportunistic Gram-negative pathogens such as *Pseudomonas aeruginosa*, *Klebsiella pneumoniae*, and *Acinetobacter baumannii* are becoming increasingly difficult to treat, as antibiotic resistance levels have risen alarmingly over recent years [5,6]. Pandemic viral outbreaks notwithstanding, this gradual blunting of weapons against clinically-relevant pathogens is arguably the most pressing challenge faced in infection biology today [5,6,7]. Already, a number of untreatable infection cases have been reported anecdotally and there is a risk of returning to pre-antibiotic era mortality rates from currently treatable infections [7].

*P. aeruginosa* is a ubiquitous opportunist, able to cause infections in plants, invertebrates, and vertebrate species [8]. In humans, it does not usually present a major burden for immunocompetent people, but can still cause eye, ear, toe, and severe burn wound infections. Importantly, it is recognised as a major nosocomial pathogen, particularly problematic in (ventilator-associated) pneumonia as well as chronic lung infections in cystic fibrosis patients [9,10,11]. In terms of resistance mechanisms, the loss of OprD-mediated resistance to carbapenems, the hyperexpression of the AmpC beta-lactamases, and the de-regulation of efflux pumps commonly arise in *P. aeruginosa* infections [2,4,12]. In the absence of new antibiotics available in the clinic in the very near future, research has started to look at anti-virulence drugs in an “if you cannot beat them, placate them” approach [4,13]. A plethora of lab-based studies, often using plant-based extracts, have shown potentially promising results [14], but pre-clinical testing is not straightforward, as the normal gold-standard read-out for testing drugs for clinical use against *P. aeruginosa*—that of bacterial killing—does not apply [4,15].

One obvious consequence of heightened resistance to antimicrobials is that bacteria are often treated at below their minimum inhibitory concentration (MIC). However, even below inhibitory concentration, the antibiotics will likely affect bacterial physiology and virulence. Moreover, the question of how much of an antibiotic’s bactericidal effect is due to its direct target effect or its broader physiological impact has been a topic of considerable debate over the past decade or so [16,17,18,19].

We were therefore interested in reviewing the current knowledge of pseudomonal virulence and how it is impacted by sub-MIC antibiotics. In this review, we briefly introduce the virulence determinants in *P. aeruginosa* and their regulation. We summarise relatively recent findings regarding the importance of surface attachment and illustrate the link between physiology, virulence, and resistance. Finally, we provide an overview of the literature on how sub-MIC antibiotic exposure impacts virulence and suggest improvements to *in vitro* models to study antibiotic–virulence factor interactions. We have limited the scope of the review to antibiotics and have not looked at papers on phytochemicals and their use as adjuvants, as most of these are not currently in clinical use [13].

## 2. Virulence Factors in *Pseudomonas aeruginosa*

As a prime opportunist, *P. aeruginosa* has a wide range of virulence factors that have different activities against different hosts and competitors [8,20,21,22]. A key challenge in reviewing changes of virulence in *P. aeruginosa* is that it is context-dependent, multifactorial, and combinatorial [23] and can therefore be quantified in several different ways.

In human hosts, several virulence factors have been identified. *P. aeruginosa* possesses an array of structurally and mechanistically distinct protein secretion systems [24] whose tasks also vary based on the competitor. For example, the type-2 secretion system (T2SS) secretes virulence factors into bacterial surroundings. Some, such as the *lasA-* and *lasB-*encoded proteases, act outside of cells, while others, such as ExoA, an inhibitor of eukaryotic protein synthesis, contain a receptor-binding domain that facilitates uptake into eukaryotic cells [24,25]. In contrast, the type-3 secretion system (T3SS) requires bacterial cell–host cell contact. It acts like a molecular syringe, injecting effector proteins directly into the host cell, and is arguably the most important protein secretion system for acute infection [26]. Effector proteins differ between strains of *P. aeruginosa,* with invasive strains using a slightly different complement of T3SS-secreted exotoxins to acute, cytotoxic ones [27,28]. Invasive strains lack the toxin ExoU, a phospholipase that leads to a rapid loss of cell membrane integrity and necrosis; instead, they use ExoS, a bi-functional toxin with GTPase-activating protein (GAP) and adenosine diphosphate ribosyl transferase (ADPRT) activity, which leads to cytoskeletal disruption and apoptotic cell death [26]. Interestingly, the type-6 secretion system (T6SS), for which three differentially regulated subtypes (H1, H2 and H3) have been described, is involved in inter-bacterial competition as well as virulence towards eukaryotes [29,30]. H1-T6SS is mainly involved in inter-bacterial competition, with virulence directed against other T6SS-positive species, while H2 and H3-T6SS exhibit anti-eukaryotic virulence and are important for the cell invasion demonstrated by ExoU-negative strains [31].

In addition to proteins, *P. aeruginosa* excretes small-molecule virulence factors. Hydrogen cyanide has been shown to be produced in microaerobic surroundings of the CF lung, where it blocks host cell oxidative phosphorylation [32,33], with *P. aeruginosa* surviving due to its own cyanide-insensitive oxidase [34]. Further, molecules with clear physiological function can have direct detrimental effects on host cells and/or competing bacterial and fungal species. Examples include the siderophores, pyoverdine and pyochelin [35]; pyocyanin, a redox-active pigment, which disrupts the host cells’ oxidative stress response while also impairing glutathione synthesis [36,37]; other redox-active phenazines [38]; and quorum-sensing (QS) molecules [39,40].

A further delivery system for virulence factors is outer membrane vesicles (OMVs) [41]. These nanosized proteoliposomes can carry various cargoes from nucleic acids to toxins [42]. OMVs are often produced as part of a stress response [43,44], but several studies have shown the active packaging of virulence-encoding DNA during the exponential phase [45], suggesting a more complex role in cell-to-cell interaction. OMVs facilitate the delivery of several virulence factors simultaneously, directly into the cytoplasm of host cells [41,42]. Such virulence factors include alkaline phosphatase, haemolytic phospholipase C, the toxin Cif, and cytotoxic alkyl quinolones [41,46]. Likewise, alkyl quinolones acting as QS molecules are also trafficked between bacteria in OMVs [47,48]. OMVs also aid antibiotic resistance by facilitating the intra- and inter-species transfer of antibiotic resistance genes [45] and of antibiotic-degrading enzymes, such as β-lactamase [41].

## 3. Regulation of Virulence in *P. aeruginosa*

**Figure 1:**
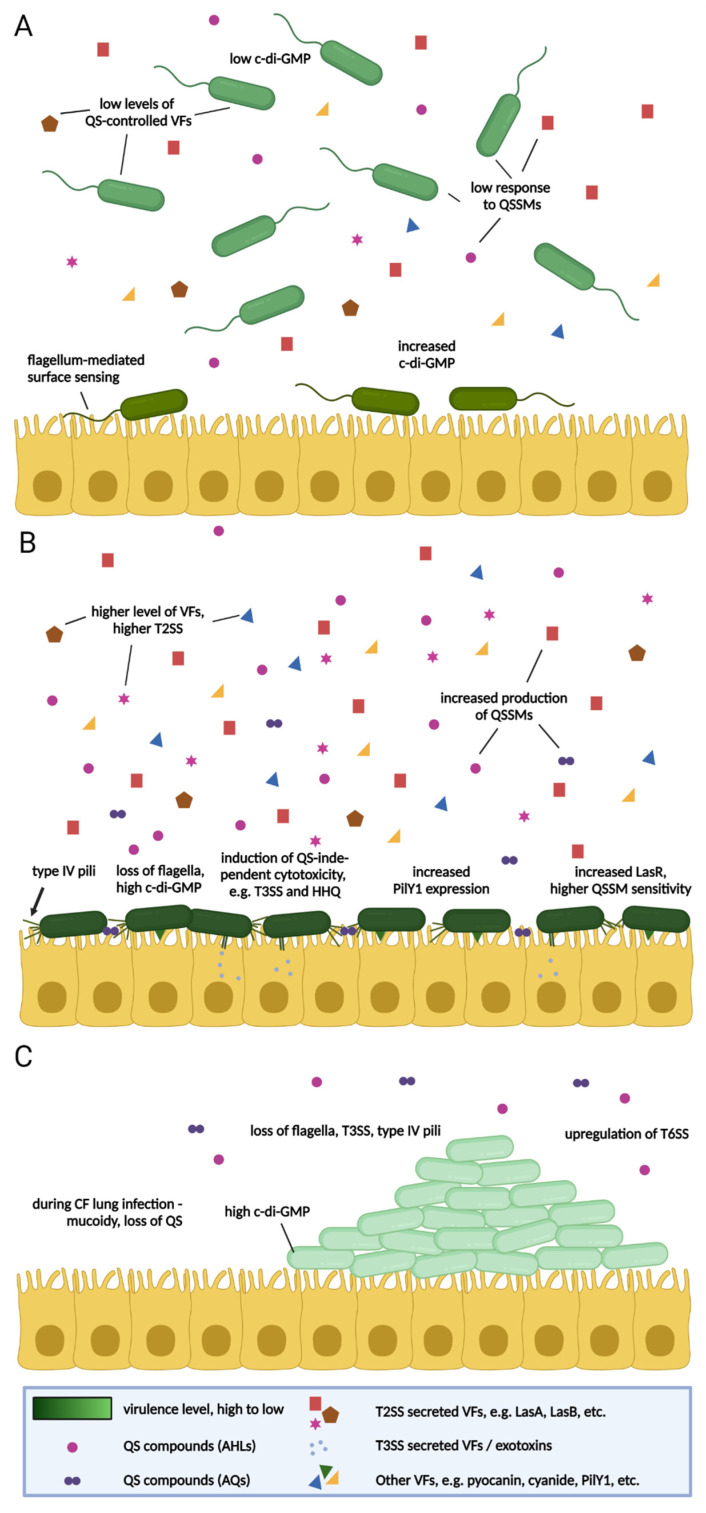
(**A**) Planktonic, free-swimming cells are flagellated, have low c-di-GMP, and, as a result of low sensitivity to QSSMs, exhibit low virulence even when a quorum is reached. Upon encountering a host cell surface, flagellum-mediated surface sensing leads to a rapid increase in c-di-GMP, although virulence remains low in reversibly attached cells. (**B**) Once cells commit to a surface and become irreversibly attached, virulence is further induced via type IV pili-mediated cAMP production. Vfr binds cAMP, activating the transcription of Vfr-regulated virulence genes, including those of the T2SS and T3SS. Virulence factors secreted by the T2SS include the elastases LasA and LasB, the protease, alkaline protease A, exotoxin A, and pyocyanin, while the T3SS secretes various cytotoxins directly into host cells. Vfr also regulates the LasR QS system, as well as the adhesin, PilY1, such that both LasR and PilY1 are up-regulated upon surface attachment. Surface-attached cells demonstrate increased sensitivity to the *las* QSSMs, causing LasR targets to be more strongly induced. LasR and PilY1 are also important for mediating surface-induced cytotoxicity via their effect on the *pqsABCD* operon and production of the AQs, including HHQ. PilY1, as well as the Wsp surface-sensing pathway, lead to signalling cascades that result in increased c-di-GMP. (**C**) Eventually, once c-di-GMP is sufficiently high, surface-committed cells start to form microcolonies and biofilms. At this stage, the bacteria lose their motility, down-regulate, or lose many of their host-directed virulence mechanisms, such as the T3SS and the *lasR* QS system, and upregulate the T6SS, important for inter-bacterial competition. Figure created on BioRender.com.

This arsenal of virulence factors is regulated by a complex network integrating bacterial environment and physiology [2,49,50,51]. In planktonic cells (see Figure 1), many of the virulence factors of *P. aeruginosa* are chiefly under the control of QS [49]. QS is a process by which bacterial cells communicate with each other through the production and detection of QS signalling molecules (QSSMs)/autoinducers [52]. Each QS system comprises synthase gene(s), which control the production of the autoinducers, and a receptor gene, which controls their detection [52]. At low cell densities, the synthase gene is transcribed at a basal level and the resulting autoinducers stay below threshold detection level. The accumulation of autoinducers in the extracellular environment due to increases in cell number leads to a threshold (“quorum”) concentration being reached. The autoinducers are then recognised by the cognate receptors, which function as transcriptional regulators, inducing the expression of target genes. These target genes include the synthase gene, thereby creating a feed-forward loop for the production of the signalling molecule [39].

Currently, there are four known systems that make up the QS network in *P. aeruginosa*—the *las*, *rhl*, *Pseudomonas* quinolone signal (PQS) and Integrated Quorum-Sensing Signal (IQS) systems [39,49,53,54]. Their signalling molecules are diverse in chemical nature. Both the *las* and *rhl* systems use acyl-homoserine lactones (HSLs), namely 3-oxo-dodecanoyl-HSL (3oC12-HSL) and butyryl-HSL (C4-HSL), respectively. The *Pseudomonas* quinolone signal (PQS) strictly refers to 2-heptyl-3-hydroxy-4-quinolone, although a number of structurally related alkyl quinolone (AQ) compounds with specific biological functions have been discovered [55,56]. Finally, IQS (2-(2-hydroxyphenyl)-thiazole-4-carbaldehyde), particularly important during phosphate limitation [53], has been suggested as the newest member of the QS signal family in *P. aeruginosa*, but its biosynthesis and regulation have been a topic of recent debate [57,58,59]. Overall, it is clear that QS is an extremely important regulator of gene expression and it has been suggested that up to 10% of all genes in the genome of *P. aeruginosa* are under QS control [60]. This includes a number of virulence factors, e.g., elastase, pyocyanin, and cyanide. Conversely, the T3SS is repressed by QS [49,61].

This “classic” model, placing a high degree of importance on the QS regulation of virulence, largely arose from studying bacteria during planktonic growth and has recently come under increased scrutiny [20,62,63]. This is because although this mode of growth lends itself to re-creation in the laboratory, it may not be the most reflective model of *in vivo* conditions, where bacteria exist in a mix of planktonic, surface-attaching/-attached, and biofilm states (Figure 1).

## 4. The Importance of Surface Attachment

Recent studies suggest that cells in a planktonic state continue to exhibit relatively low virulence even once QS is activated [64]. It is not until they attach to a surface that virulence is fully induced, provided bacterial cell density is sufficiently high. In this way, the two signalling pathways of QS and surface sensing effectively function as a logic gate, “AND”, initiating (host-independent) virulence activation—an energy-expensive behaviour—only once the bacteria can sense that it will be effective in killing the host cells [64].

The mechanism by which surface-attached cells induce virulence towards host cells is thought to be multifactorial [46,64]. LasR is an important regulator of surface-attached virulence: upon surface attachment, *lasR* expression is up-regulated and the induction of LasR targets in response to the *las* QSSM, 3oC12-HSL, is stronger than in planktonic bacteria [65]. Although a full understanding of the mechanisms by which *P. aeruginosa* senses the presence of a surface and induces virulence is yet to be determined, flagella, type IV pili, the cell surface adhesin PilY1, and minor pili have all been identified as playing a role [46,63,64,66,67]. PilY1, which is up-regulated upon surface contact, seems to be important for initiating acute, cytotoxic virulence in surface-attached cells (as does LasR) [46,64]. This cytotoxicity appears in part to be driven by LasR- and PilY1-dependent activation of the *pqsABCD* operon and the subsequent production of the PQS precursor, HHQ. HHQ has been found to be directly cytotoxic to animal cells and the packaging of this AQ by *P. aeruginosa* into OMVs increases delivery efficiency into host cells, furthering toxicity [44,46].

Recent work by Laventie et al. suggests that *Pseudomonas* employs an astonishing bet-hedging strategy upon encountering a host surface. The regulatory circuits governing surface attachment and virulence regulation involve an interplay of two-component systems (including the RetS, LadS, and GacA systems) and non-coding regulatory small RNAs (*RsmY*, *RsmY*, and *RsmZ*) and are connected by the small molecule messenger, cyclic-di-GMP [2,63,68]. When meeting a host cell, it appears that flagellum-mediated mechanical stimuli may be the mechanism by which *P. aeruginosa* senses the cell surface [63]. This causes cyclic-di-GMP levels to rapidly rise, which in turn leads to active FimW accumulating at the cell poles where it stimulates the assembly of type IV pili, necessary for surface attachment. Asymmetric division of the surface-bound cell then follows, with two cell types arising: a flagellated, non-piliated, planktonic cell (with low cyclic-di-GMP levels), which is free to spread, and a piliated, surface-bound cell, with high cyclic-di-GMP levels, which is highly virulent and attacks the host epithelium. Type IV pili mediate virulence not only through their mutually dependent interaction with the T3SS [69], facilitating the injection of acutely cytotoxic type-3 exoenzymes into host cells, but also through continued surface sensing, causing the activation of signalling pathways, which leads to increases in cAMP and the induction of virulence genes [63].

This provides an important update to the view where *P. aeruginosa* exhibits binary modes of growth: a more virulent planktonic mode and a less virulent biofilm mode, with surface attachment regarded as the first step towards virulence reduction [2]. Instead, virulence would seem to be more nuanced, with an interim stage of hypervirulent surface attachment and planktonic virulence likely of lower importance than previously thought [68] (Figure 1).

## 5. Virulence in *P. aeruginosa*—Context and Time

*P. aeruginosa* has the ability to cause acute infection—for example, on burn wounds—as well as chronic infections, most prominently in the lungs of CF patients, where clonal infections have been shown to last decades [70,71,72,73]. The virulence factors employed across an infection event are regulated within a dynamic system so that inputs into a particular stage of the system not only affect one regulatory cascade and its output but also affect the system as a whole.

CF lung infections have been studied extensively due to the availability of longitudinal, often clonal isolates and patient-specific lineages that have evolved in the lung for decades [71,73]. Studies on these isolates have shown that after initial infection, the bacteria by and large switch to a more chronic infection phenotype. The bacteria lose or downregulate “offence” behaviours that provide active, host- or competitor-harming virulence—such as the T3SS—and also frequently lose their QS ability due to mutation in *lasR* [71,74], while increasing “defence” behaviours—such as losing their immunostimulatory flagellum—that aid survival [75,76]. Later CF isolates routinely exhibit lower virulence in model systems such as the *Galleria mellonella* infection model [77].

A hallmark of this switch is the over-production of alginate in the mucoid morphotype of *P. aeruginosa*, which is almost pathognomic for the CF lung [78]. Mucoidy is usually caused by a mutation in *mucA*, the gene encoding a pseudomonal anti-sigma factor [78,79]. MucA normally sequesters the alginate synthesis-controlling sigma factor AlgU and is proteolytically cleaved, e.g., in the event of cell-wall stress [80]. Mucoid isolates are associated with a worsening prognosis in CF patients but consistently exhibit—in *in vitro* studies at least—lower peak production of virulence factors such as hydrogen cyanide, elastase, and pyocyanin, potentially due to the differential regulation of the PQS system [78]. Interestingly, the constitutive synthesis of alginate comes at a physiological cost, as mucoid isolates of *P. aeruginosa* are more susceptible to osmotic stress [81,82,83].

Several patient-to-patient transmissible isolates such as the Liverpool epidemic strain, LESB58 [84], or the Danish strains, DK1 and DK2 [71], have been described. These isolates have led to infections in several CF patients and form a valuable resource to understand between-patient and within-patient evolution. For the DK1 and DK2 strains, it was concluded that the bacteria have evolved to a CF lung-adapted phenotype with limited in-patient changes during infection [71,74]. Somewhat conversely, for the LESB58 isolate, sub-MIC antibiotic exposure has been suggested to lead to the diversification of virulence determinants *in vitro* [84]. The same centre reported a high degree of diversification in recovered isolates during or shortly after pulmonary exacerbations compared to standard visits [85]. As aggressive antibiotic chemotherapy is a necessity during exacerbations, this highlights the interplay between antibiotic therapy, bacterial community dynamics, and virulence.

In all infection settings, virulence is context-dependent and a reaction to several external inputs which range from the direct consequences of nutrient limitation to intracellular metabolic flux changes that bring about changes in virulence. With regards to nutrients, phosphate limitation has been identified as a clinically important factor linked to post-surgical sepsis [86] and phosphate (as well as iron) depletion triggers the activation of virulence factors including proteases, cyanide, and rhamnolipids [2].

Oxygen availability greatly influences pseudomonal physiology and, by extension, virulence, as well as antibiotic resistance. *Pseudomonas* can employ several different metabolic pathways to survive in microaerobic and anaerobic conditions. It can maintain oxidative phosphorylation by using nitrate as a terminal electron acceptor in place of oxygen [87,88,89]. It can also grow anaerobically on arginine, converting arginine to ornithine and potentially using it as a nitrate donor [90], and it can further survive anaerobically using a fermentative pyruvate pathway, though this pathway does not support growth [91,92]. In low O_2_, high NO_3_ conditions, *Pseudomonas* also triggers biofilm production and changes to its membrane composition [93], both of which aid survival by providing protection from environmental stresses, including antibiotics. Interestingly, QS and QS regulators play a role in the regulation of denitrification, in which nitrate reduction is the first step. Both the *las* and *rhl* QS systems repress the transcription of denitrification genes, with regulation by the *las* QS system being dependent on the *rhl* QS system [94]. PQS represses the activity of three of the four enzymes in the denitrification pathway but increases nitrite reductase [94]. This leads to the increased production of nitric oxide (NO), a volatile intermediate of denitrification [90]. NO causes biofilm dispersal [95], is toxic in *rhlR* mutants [96], and contributes to the regulation of T3SS expression and other virulence factors [97,98], further highlighting the link between physiology and virulence. This link is also evidenced in the production and excretion of hydrogen cyanide, a toxin that blocks the cytochrome c oxidase, which is under partial QS control and upregulated (via the regulator, ANR) under low oxygen tension [33].

Carbon source availability has been shown to directly impact virulence factor production and physiology. In rich media, the carbon source hierarchy is a tightly controlled process [99], governed by the Crc and Hfq regulators in conjunction with the small RNAs, *CrcY,* and *CrcZ* [100,101]. Crc activity represses pyocyanin production [102] and promotes T3SS expression [103]. For arginine, Luckett et al. (2017) established the importance of functional arginine transporters for virulence in mice with infected burn wounds [104], while another study (which used a similar model) went on to highlight that the amount of arginine is important. Arginine supplementation, resulting in high arginine availability, was shown to suppress swimming motility, attenuate virulence, and prolong survival [105], while low arginine availability was associated with higher virulence. High arginine also represses swarming motility *in vitro* via the modulation of c-di-GMP and AQ signalling [106]. In addition, Anderson et al. (2008) identified arginine as crucial for the formation of biofilms in bacterial–epithelial cell interaction models [107]. A potential mechanistic link could be a rare arginine codon in *rhlR* that might lead to reduced levels of RhlR in low arginine backgrounds [108].

Virulence and antibiotic tolerance are often intrinsically linked via metabolism and physiology, in particular redox state. Advances in metabolic profiling and flux analysis have shed some light on how pathway activity links both virulence and resistance to physiology. Due to the lack of a functional phosphofructokinase, central carbon metabolism in pseudomonads does not flow through a canonical Embden–Meyerhof–Parnas pathway. Instead, it is organised into two interconnected cycles, with hexose metabolism occurring in the newly discovered EDEMP cycle [109,110]. The EDEMP cycle is connected to the TCA at the phosphoenolpyruvate/pyruvate/lactate node. Several studies have now shown that antibiotics contribute to oxidative stress (e.g., [16,111]), and carbon sources that lead to the activation of oxidative metabolism have been associated with higher bacterial susceptibility [112].

Conversely, it has also been shown that redox-active virulence factors contribute to resistance. In *Pseudomonas*, phenazines—redox-active pigments, capable of electron transfer, that include the known virulence factor, pyocyanin [36]—are coupled to the respiratory mechanism, with carbon-source dependent levels [38]. In a simple, elegant study, Zhu et al. (2019) showed that antibiotics promote pyocyanin release and—perhaps more importantly—that pyocyanin promotes antibiotic tolerance not just in *P. aeruginosa*, but also in other Gram-negative bacteria [113].

**Table 1:** Summary of the impact of sub-MIC antibiotic exposure on *P. aeruginosa* virulence factor production. The colour of the reference indicates the direction of change. Red indicates decreased expression/production, green indicates increased expression/production and blue indicates unchanged or bi-directional and/or concentration-dependent expression/production of virulence factors. The bar charts are normalised to 100% of all studies identified for the antibiotic.

	Pyocyanin	Pyochelin	Pyoverdine	Las A	Elastase	Swimming	Swarming	Twitching	Adherence	Biofilm	Vesicle form.	T3SS	T6SS	Alginate	Phospholipase C	Alk. Protease	Exotoxin A	3oC12-HSL	C4-HSL	PQS family	Setting—A: Animal; B: Batch Culture; C: Cell culture; CI: Clinical	Length—S: Short (24 h); I: Intermediate (1–4 d); L: Long (>4 d)	Other Competition +/− Cooperation +/−
Aminoglycosides																							
Kanamycin	[114]		[114] [115]	[114]		[114]	[114]			[116] [114]		[116]	[116]	[115]				[114]			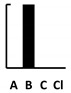	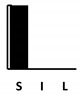	
Gentamicin	[114,117,118]		[114,115,118]	[114]	[119] [120,121,122,123]	[114]	[114,124] [125]	[124]	[126,127,128] [122,126,129]	[116] [114,118,124,130]	[119]			[115,121,127,131,132]	[119][123,133]	[119][118,120,133]	[121,122,123]	[114,118,124,134]	[124,134]		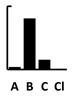	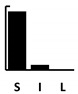	Comp. [135,136] Cooperation [117] *in vitro* virul. [133]
Amikacin	[114]		[114]	[114]		[114]	[114]		[137]	[114]				[132]				[114,134]	[134]		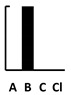	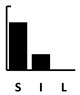	
Netilmicin	[114]		[114]	[114]		[114]	[114]		[138]	[114]				[132]				[114]	[134]		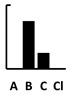	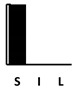	
Paromomycin	[114]		[114]	[114]		[114]	[114]			[114]								[114]			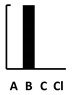	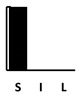	
Neomycin B	[114]		[114]	[114]		[114]	[114]			[114]								[114]			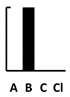	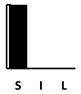	
Tobramycin	[139,140] [141]	[107]	[107,115]	[141]	[120,121,142,143,144]	[145]	[145] [140,141]		[127,129,146] [147]	[116,145,148] [116] [139,140,141]	[149]	[107,143,144] [145]	[149]	[107] [132] [115,121,127]	[143]	[141] [120,121,149]	[143]	[148] [142,149]	[148] [140]	[148] [107]	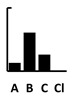	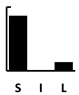	*in vivo* virul. [143,144,150] *in vitro* virul. [107,151]
Streptomycin	[139]		[115,139]		[139]	[139]	[139]	[139]		[139]				[115,132,139]	[133,139]	[133]		[139]	[139]		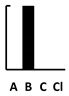	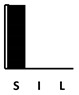	Haemolysis [139] *in vitro* virul [133]
**Beta-Lactams**																							
**Penicillins**																							
Carbenicillin			[115]		[121,122]				[122,129]					[115]			[121] [122]				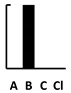	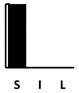	Competition [135] [136]
Aztreonam														[132]							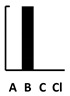	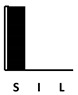	
Ticarcillin									[129]					[132]	[133]	[133]					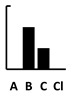	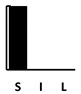	*in vitro* virul. [133]
Ampicillin	[152]			[152]	[152]							[152]		[152]		[152]	[152]		[152]		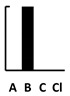	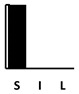	
Piperacillin/tazobactam						[153,154]		[153,154]	[153,154]	[154]											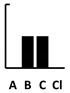	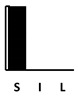	
**Carbepenems**																							
Meropenem									[129]												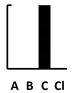	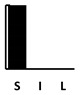	
Imipenem	[155]				[155]	[153]		[153]	[129,153]	[156]				[132][156]				[155]	[155]		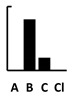	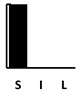	
**Cephalosporins**																							
Ceftazidime	[84,155,157]		[115]		[123,143,155,157,158] [121,142,144]		[145,157,159]	[145,159,160]	[129,146] [137]	[145,157,159,160,161]		[143,144]		[132] [115]	[123,143]	[157,158]	[123,143] [121]	[142,145,155,157,158,159]	[142,145,155,157,158,159]	[157]	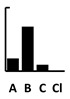	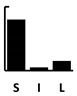	*in vivo* virul. [143,144,150,157]
Cefotaxim									[161]						[133]	[133]					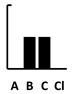	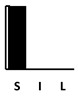	*in vitro* virul. [133]
Ceftriaxone									[162]												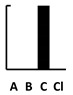	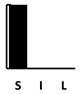	
Cefepime	[155]				[155]				[147]									[155]	[155]		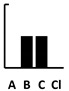	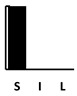	
Cefsulodin									[128]												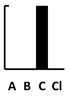	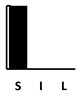	
**Macrolides**																							
Azithromycin	[152] [163,164,165,166]		[166]	[167] [152]	[168][123,152,158,164,167,169,170,171]	[167,169,172,173]	[124,125,163,165,166,173]	[124,165,167,173]	[174,175]	[124,165,166,173,176,177,178]		[158,167]		[131,164,177,178] [152]	[123,172]	[158,165,170] [152]	[123] [152]	[124,158,164,168,171,173,176,179]	[124,158,165,171,173,176,179]		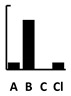	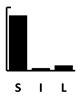	*in vivo* virul. [150,168] [164,172,173] *in vitro* virul. [167]
Clarithromycin					[169]	[169]	[125]		[147]					[131]							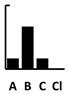	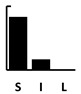	*in vivo* virul. [150]
Erythromycin	[165]				[123,169,180]	[169]	[125,165]	[165]	[181] [182]	[183] [165,178]				[131,178] [132]	[181] [123]		[123,181]	[183]	[165] [183]		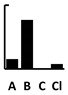	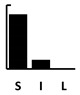	*in vivo* virul. [150] [181,184]
Roxithromycin					[123]					[183]				[132] [185]	[123]		[123]	[183]	[183]		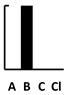	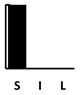	
**Tetracyclines**																							
Tetracycline	[152]	[186]		[152]	[152,186]	[145]	[125,145]			[116,145] [139]		[145] [152] [186]		[152]		[152] [187]	[152][186]		[152]		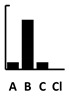	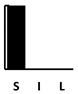	*in vivo* virul. [145]
Doxycycline	[188]				[188]		[188]			[188]											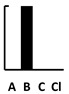	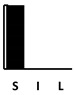	*in vivo* virul. [188]
**Fluoroquinolones**																							
Ciprofloxacin	[189] [190]	[191]	[115]		[143,144,158,191,192]	[193] [145,191] [194]	[194] [145,159,191,195]	[145,159,191,194,196]	[126,129,137,147,197]	[145] [159,192] [193]		[143,144] [145]		[191] [115,132]	[143,191]	[158,191]	[143]	[192,194,196] [145,158,159,191] [193]	[145,158,159,191] [193]	[193]	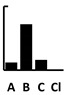	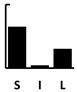	*in vivo* virulence [143,144,198]
Enoxacin									[199]					[132]							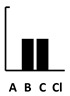	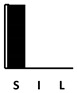	
Lomefloxacin									[199]												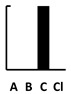	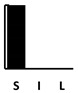	
Norfloxacin										[200]				[132]							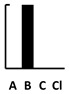	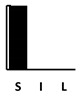	
Ofloxacin					[123]									[132]	[123]	[123]					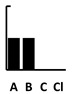	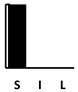	*in vivo* virul. [150]
Perfloxacin														[132]							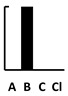	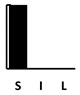	
**Cationic peptides**																							
Polymyxin B									[128]												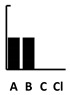	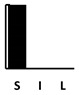	competition [136]
Colistin	[201] [84]					[201]	[201]	[201]	[201]									[201]	[201]	[201]	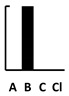	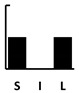	
**Others/Non-categorised**																							
Trimethoprim									[197]												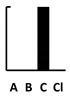	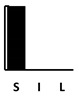	
Sulfamethoxazole										[183]								[183]	[183]		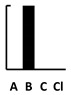	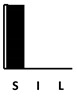	
Chloramphenicol							[125]		[128]					[131]							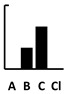	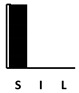	
Rifaximin (Ansamycin)	[159,190]																				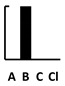	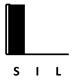	
Vancomycin(Glycopeptide)	[152]			[190]	[152]	[152]	[152]					[190]		[190]		[190]	[190]		[190]		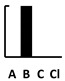	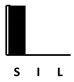	
Nalidixic acid(Quinolone)														[132]							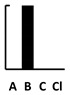	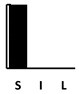	
Clindamycin(Lincasamide)									[202]					[131]							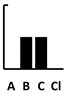	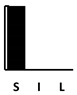	

Fatty acids are utilised via beta-oxidation and funnel into the TCA cycle as acetyl-CoA. To use these two-carbon units for growth rather than just ATP generation, *P. aeruginosa* turns to the glyoxylate shunt (GS), a bypass pathway that cleaves isocitrate to form succinate and glyoxylate, which, with another molecule of acetyl-CoA, forms malate, a canonical TCA cycle intermediate. Several studies have identified the activation of the GS *in vivo* or in mimicking settings [20,62,203] and revealed the importance of fatty acid utilisation for infection [62,204,205]. Activation of the GS is also linked to the activation of the T3SS [206,207]. It is therefore clear that *P. aeruginosa*, with its large metabolic capacity and tightly controlled compound uptake [99,100,101], will alter its virulence and resistance profile in response to the environment. This, in turn, means that adjusting media formulation *in vitro* to accurately mimic the site of infection is crucial [20].

## 6. The Impact of Sub-MIC Antibiotics on Virulence in *P. aeruginosa*

To date, most studies undertaken to investigate the impact of sub-MIC antibiotics on virulence in *P. aeruginosa* have been performed over a short term (<24 h antibiotic exposure) on planktonic bacteria cultured in rich media and in the absence of other cells, with virulence measured via *in vitro* assays (Table 1). By and large, these planktonic studies have shown that sub-MIC antibiotic exposure causes a reduction in most secreted virulence factors, including pyocyanin; pyochelin; pyoverdine; LasA and LasB proteases; swimming, swarming and twitching motility; adherence; biofilm formation; the T3SS; alginate; phospholipase C; alkaline protease; exotoxin A; LasR- and RhlR-binding QSSMs; and virulence factors of the PQS family (Table 1). Results from these studies have then informed the consensus that most sub-MIC antibiotics will reduce the *in vivo* virulence of *P. aeruginosa*, certainly in acute infection scenarios.

However, while it is the case that free-swimming, planktonic cells in nutrient-rich environments certainly exist *in vivo*, e.g., in the lungs of CF patients during exacerbations [71,208], we now know that *P. aeruginosa* exists in various states of motility, which include free-swimming, surface-attaching and -attached, as part of a microcolony, or contained within a biofilm [46,63,64]. In addition, *in vivo*, pseudomonads will encounter other bacteria and host cells and will come across varying nutrient availability and other stresses, all factors that affect physiology and virulence [209,210].

Therefore, we chose to focus the review on discussing the effect of sub-MIC antibiotics on the virulence of *P. aeruginosa* in these different contexts, with the aim of seeking to understand whether the (largely inhibitory) effects witnessed when the planktonically grown bacteria are exposed to sub-MIC antibiotics over the shorter term can realistically be expected to deliver the same benefits *in vivo*. Given that in chronic infection scenarios, the bacteria can evolve in the presence of sub-MIC antibiotics over long periods, we have split our review into two sections, based on antibiotic exposure times. We firstly cover short- and intermediate-term (“shorter-term”) studies, in which *P. aeruginosa* was respectively exposed to sub-MIC antibiotics for less than 24 h and between 1 and 4 days, with the majority of these studies being short term. We then move on to looking at longer-term studies, in which *P. aeruginosa* was evolved in the presence of sub-MIC antibiotics for at least five days.

### 6.1. Shorter-Term Studies

#### 6.1.1. *In Vitro* Studies

##### Mode of Growth

As mentioned above, the majority of *in vitro* studies to date have used planktonically grown *P. aeruginosa*, even though the bacteria exhibit a variety of growth modes *in vivo* [68], with surface attachment known to be important for virulence induction [46,64]. In particular, recent evidence suggests that the bacteria switch between planktonic, surface-attaching/-attached, and biofilm lifestyles more fluidly than previously thought [68]. This section, therefore, looks at what we know about the effect of sub-MIC antibiotics on the pathogen’s virulence in these different modes of growth.

##### Biofilm

For studies involving pre-formed biofilms, findings have been antibiotic-dependent. Sub-MIC imipenem appears to further promote biofilm growth—increasing alginate production, biofilm thickness and biomass, and reducing motility [156]. In contrast, sub-MICs of several aminoglycosides [114,139], macrolides [178], and tetracycline [139] cause biofilm dispersal. Surface attachment and quorum sensing, both important for virulence and for biofilm formation [64], are also differentially affected. In pre-formed biofilms, exposure to the sub-MICs of several fluoroquinolones reduces the adherence of *P. aeruginosa* [211], but sub-MIC levofloxacin, a newer fluoroquinolone, has been found to upregulate QS-related genes [212].

The sub-MIC tobramycin treatment of mature *P. aeruginosa* biofilms established on CF bronchial epithelial (CFBE) cells has been found to reduce cytotoxicity without lowering bacterial numbers [107]. Transcript levels for various factors known to be important for virulence were downregulated, including those involved in phenazine synthesis, hydrogen cyanide synthesis, pyoverdine activity, and iron chelators [107]. Mechanistically, however, the evidence points to reduced AQ production and T3SS secretion [46,107]. The genes involved in AQ biosynthesis (*pqsABCDE*) were downregulated, potentially due to increased transcription of *algR* [107]. *algR* encodes AlgR, a positive regulator of alginate biosynthesis, but it also indirectly reduces *pqsABCDE* expression [46]. *algR* was found to be upregulated without a corresponding increase in the transcription of genes involved in the alginate synthesis pathway, such as *algD.* Reduced *pqsABCDE* expression would lead to the decreased production of HHQ, an AQ found to be directly cytotoxic to mammalian cells [46]. Genes involved in the regulation of the T3SS were also affected. *dnaK,* which has been shown to be important for type-3 effector secretion [213], was downregulated [107], while *mgtE*, an inhibitor of the T3SS activator ExsA [214], and *algU*, which encodes the RNA polymerase sigma factor AlgU, a negative regulator of the T3SS [2], were upregulated [107]. Interestingly, the transcriptional suppressing effects of tobramycin on *pqsA* (whose gene product, PqsA, is required for initiating AQ production) and *dnaK* were only observed when biofilms were grown on the (biotic) epithelial cell surface, but not on abiotic surfaces [107]. Furthermore, there was little correlation between the genes that were affected by tobramycin in biotic biofilms, abiotic biofilms, or planktonic cell cultures. Together, these findings highlight the importance of using a variety of relevant models.

##### Surface-Attached

Sub-MIC tobramycin has also been found to have anti-virulence effects in surface-attached *P. aeruginosa* cells. In another study by Anderson et al., sub-MIC tobramycin was investigated for its ability to inhibit biofilm formation by *P. aeruginosa* on CFBE cells. The authors used the same model as used in their 2008 study, but this time added the antibiotics before the biofilm had formed. Although at the concentration used (4 µg/mL), the antibiotic failed to inhibit biofilm formation, it did act to prevent bacterially mediated cytotoxicity towards the CFBE cells [151].

##### Co-Operation between *P. aeruginosa* Strains

The phenomenon of “social cheating”, i.e., bacterial members that take advantage of the public goods produced by co-operators without having to incur the metabolic expense of production, has been well described in *P. aeruginosa* [215]. However, few studies have sought to determine the effect sub-MIC antibiotics have on co-operative behaviour in *P. aeruginosa*, with most studies instead employing the use of a single strain. Of the studies that have been undertaken, contrasting findings have emerged. In 2010, Köhler et al. investigated the effect of azithromycin (AZM) on these interactions in media that necessitated the production of *lasR*-controlled proteases to maximise growth. In the absence of AZM, *lasR* mutants exhibited a reduced growth rate (by about 50%) compared to the wild type in monoculture, but exhibited a fitness advantage in mixed populations, conforming to the expectations of wild type–social cheater interaction [168]. With AZM, wild types showed a greater decrease in growth rate than *lasR* mutants in monoculture, while in mixed cultures, the *lasR* mutants’ fitness advantage decreased with increasing concentrations of AZM, suggesting that AZM had suppressed QS-controlled exoproducts [168]. In contrast, a similar study using the partially QS-controlled siderophore pyoverdine as the public good in iron-limiting conditions found that the proportion of cheaters greatly increased under sub-MIC gentamicin exposure [117]. This was likely due to constitutive investment in pyoverdine production having lowered the ability of the co-operating producers to cope with antibiotic stress in the presence of cheating non-producers [117]. The two studies neatly highlight that the cost/benefit balance of public good production can tip in the presence of antibiotic stress to favour either (virulent) producers or (less virulent) non-producers.

##### Competition between *P. aeruginosa* Strains and Other Bacteria

Recognising that *P. aeruginosa* exists as part of a polymicrobial community in various infection scenarios, more and more studies are now being undertaken to investigate the impact that the existence of other bacteria has on the virulence of *P. aeruginosa* and vice versa [216]. However, although studies have been performed with MIC levels of antibiotics [217,218,219], there is a dearth of *in vitro* studies looking at how perturbations in the environment, brought about by sub-MIC antibiotics, affect this bacterial competition. Exposure to sub-MIC kanamycin has been shown to cause *P. aeruginosa* to induce the T6SS [116], which is thought to be involved in bacterial–bacterial interactions. Further studies using polymicrobial models are needed, however, to elucidate the polymicrobial dynamics in response to sub-MIC antibiotic exposure and the likely implications for overall *in vivo* virulence.

Similarly, different strains of *P. aeruginosa* may be present in an infection, at least in the early stages [220], and knowledge of the effect of sub-MIC antibiotics on this intraspecies competition is limited. A couple of early studies investigated how exposure to sub-MIC antibiotics affected pyocin production by *P. aeruginosa*. Pyocins are antimicrobial proteins that act against strains of the same, or closely related, species [221,222]. Interestingly, exposure to sub-MIC ciprofloxacin has been found to significantly upregulate the expression of genes involved in pyocin production [223]. Sub-MIC polymyxin B was found to protect *P. aeruginosa in vitro* from pyocins to which it is usually susceptible, possibly by enacting changes in lipopolysaccharide (LPS) composition [136], whilst sub-MIC carbenicillin changed the pyocin type produced by *P. aeruginosa* and protected various *Enterobacteriaceae* strains (*Escherichia coli*, *Salmonella typhi*, *Proteus vulgaris*, and *Shigella flexneri*) [135]. Sub-MIC gentamicin demonstrated the same effects [135,136]. Although the mechanisms behind these findings were not explored at the time, a more recent study helps shed some light on them. From a series of intraspecies competition experiments, Oliveira et al. (2015) discovered that pyocins initiate biofilm formation [220]. As revealed by experiments in which biofilm formation increased in mixed cultures versus monocultures, and by pairwise experiments in which one strain tended to dominate, the induction of biofilm formation was found to be a response to ecological competition, rather than a co-operative endeavour. Further experiments revealed that pyocins served as the cue, but only when they induced damage, and that, mechanistically, increased surface attachment was at least partly responsible. The authors decided to explore whether the oft-reported biofilm response to antibiotics also had its origins in responses to ecological competition. Although several earlier studies had found that sub-MIC antibiotics induced biofilm formation, many others reported the opposite (reviewed in [224]). Suspecting, from their own investigations using static biofilm assays, that the effect was antibiotic concentration-dependent, the authors employed a microfluidic device. This allowed them to perform the experiments using a gradient of antibiotic concentrations, all the way up to lethal doses, and observe how the bacteria responded. Three different antibiotics—ciprofloxacin, rifampicin, and tetracycline—were used, and in all cases, sub-lethal doses did indeed initiate biofilm formation. Furthermore, the different modes of action of the three antibiotics (the inhibition of DNA replication (ciprofloxacin), transcription (ripampicin), and translation (tetracycline)) and of the pyocins (the disruption of membrane potentials), together with a variety of culture media used in the static biofilm assays, lead the authors to conclude that the observed responses can likely be generalised. Any toxin produced by one *P. aeruginosa* strain may have the potential to initiate biofilm formation in another. By extension, it seems that any sub-MIC antibiotic capable of inducing damage is likely to have the same effect. These findings clearly have important implications for the administration of antibiotics in the clinic, particularly at the early stage of infection, and once again highlight the importance of appropriate experimental design.

#### 6.1.2. *In Vivo* Studies

Various studies have sought to employ animal models that replicate chronic *P. aeruginosa* infection, through, for example, the use of *P. aeruginosa* delivered with agar beads or in alginate [225]. While these models recreate some long-term adaptation effects, the infection is usually only established for a short time before antibiotics are administered, and extensive host tissue damage can be caused in that time. In reality, most models are therefore comparable to acute infection scenarios, although the beads impede bacterial clearance. Various experiments have been performed using animal models to investigate the effects of a shorter-term administration of sub-MIC antibiotics—mainly macrolides—on the *in vivo* virulence of *P. aeruginosa*. These have yielded some interesting findings and contributed to the understanding of the drugs’ possible mechanisms of action.

For AZM, the beneficial effects of sub-MIC treatment have been described for healthy and CF mice with *P. aeruginosa* lung infections. AZM reduces inflammation and attenuates neutrophil infiltration of the lungs [164,226,227] through reduced pro-inflammatory cytokine production [226,227]. It also significantly decreases the bacterial load [164,226,228] and the severity of the pathology (including the extent of abscesses) in the lungs [164], leading to reduced mortality [226]. Part of its mechanism of action may be explained by the destruction of biofilms through the inhibition of alginate production, as alginate content was found to be much lower in the lungs of the mice treated with AZM than in the control [164]. Increased susceptibility to stationary phase killing [164,228], increased sensitivity to serum bactericidal activity [164], and the increased killing of biofilm bacteria by neutrophils [226] may also contribute to reduced bacterial numbers. The drug has also proven effective against *P. aeruginosa* in mouse models of renal infection (pyelonephritis), leading to reduced inflammation and a lowering of bacterial numbers [173].

Interestingly, although AZM has proven beneficial when administered to mice already infected with *P. aeruginosa*, the intranasal inoculation of mice with *P. aeruginosa* that had been pre-treated with sub-MIC AZM for 24 h led to 100% mice mortality [150]. In contrast, the mice challenged with either untreated bacteria or bacteria treated with non-macrolide antibiotics did not die within the study period, nor did the mice treated with AZM after inoculation with *P. aeruginosa*. The effect was likely not mediated by bacterial exo-products, as mice challenged intratracheally with the supernatant from *P. aeruginosa* grown in the presence of sub-MIC AZM demonstrated a low inflammatory reaction compared to mice challenged with the supernatant from *P. aeruginosa* grown without the antibiotic [166,229]. A histopathological examination of the lungs revealed an influx of numerous inflammatory cells, and so it would appear that AZM pre-treatment of bacteria led to the *in vitro* supercharging of bacterial virulence and cell-to-cell toxicity (most of the mice died within 9 h), which in turn triggered a detrimental immune response [150]. In agreement with these findings, the treatment of *P. aeruginosa* with sub-MIC AZM has been shown to cause the upregulation of the T3SS [152,158,167]—a major determinant of the pathogen’s virulence, which facilitates the release of directly cytotoxic proteins into eukaryotic cells upon encounter [24]. Sub-MIC AZM has further been shown to increase the expression of the surface-exposed protein, PilY1 [170], which is important in mediating surface-associated virulence induction [46,64]. This super-charging of cell-to-cell toxicity so far seems to be unique for AZM, as mice injected intraperitoneally or intravenously with *P. aeruginosa* pre-treated with sub-MIC erythromycin demonstrated increased survival and clearance of the bacteria, compared to mice infected with untreated *P. aeruginosa* [181,184].

Clinically, AZM has been shown to promote host defences when administered early during bacterial infection, whereas it inhibits inflammation and promotes resolution in chronic infections [230]. It selectively accumulates in neutrophils and alveolar macrophages, with the intracellular drug concentration found to be as much as 2000-fold higher in neutrophils than in plasma, likely contributing to the enhanced killing of *P. aeruginosa in vivo* [231].

### 6.2. Longer-Term Studies

Compared to the shorter term, there is a relative paucity of studies looking at the impact of longer-term sub-MIC antibiotic exposure on the virulence of *P. aeruginosa*. However, such studies are important, given that in chronic infection scenarios, e.g., the CF lung or in non-healing diabetic wounds, the bacteria may evolve in the presence of sub-MIC antibiotics over long periods. Below, we cover some of the studies that have been undertaken, focusing on *in vitro* studies first before moving on to *in vivo* studies.

#### 6.2.1. *In Vitro* Studies

##### Planktonic

The fluoroquinolone ciprofloxacin (Cip) is probably the best-studied antibiotic in the context of long-term laboratory evolution. Cip is thought to exert bactericidal effects by causing the accumulation of hydroxyl radicals (subsequent to the inhibition of the target, DNA gyrase) and a resultant increase in oxidative stress [16,19]. In response to Cip-induced damage, *P. aeruginosa* activates genes involved in the SOS response [232]. Medium-term Cip exposure leads to an adaptive phenotype, which includes increased resistance [233], often mediated by mutations of the transcriptional repressor *nfxB*, leading to the overexpression of the efflux pump MexCD-OprJ, and mutations causing the overexpression of the efflux pump MexAB-OprM [234,235]. Long-term effects on virulence differ significantly from the mostly inhibitory effects of short-term exposure, which include reductions in alginate; swimming, swarming, and twitching motility; quorum sensing; and protease and/or siderophore production (e.g., [158,191,193]).

The Ciofu lab has, in recent years, investigated the effects of medium- to long-term (hundreds of generations) Cip exposure on *P. aeruginosa* in a set of elegant experiments. In a study focusing on planktonic cells [192], the authors compared the effects of continuous sub-MIC (0.1 µg/mL) Cip exposure to controls. They found that while protease and swimming motility were decreased, Cip exposure stabilised QS activity, an effect also seen by Köhler et al. (2010) in the presence of AZM. In rich media (and the CF lung), *P. aeruginosa* routinely acquires mutations in the QS regulatory circuitry (often in the *lasR* gene, which codes for LasR, the regulator at the top of the QS hierarchy in *P. aeruginosa* [49]). Such mutations are thought to provide a competitive advantage in non-stressed rich media conditions [236]. The authors suggest that the reason why *P. aeruginosa* may act to maintain a functioning QS system in Cip-exposed cultures is because the QS-controlled production of catalase and superoxide dismutase might be of higher importance as a result of Cip-mediated oxidative stress. Indeed, an earlier study found that oxidative stress strongly selects for *P. aeruginosa* strains with active QS systems that can mount a robust defence against it. In this way, the proportion of QS-null cheaters was reduced in cultures otherwise prone to invasion by cheats [237]. In subsequent studies, the authors extended their experimental design to include biofilms and a mutant of catalase (*katA*). While the planktonically grown *katA* mutant accumulated more mutations overall [238], just like in the wild types, QS was stabilised in the Cip-treated mutants and often lost in the control mutants [194]. These later studies also found that, compared to the controls, twitching motility was impaired in the Cip-evolved populations [194,196].

##### Biofilm

The group’s later studies saw pseudomonal biofilms being cultured concurrently with planktonic cells [194,196,238]. Although sub-MIC Cip exposure led to the development of resistance in both modes of growth, different patterns emerged. The resistant sub-population reached a higher proportion in biofilms than in planktonic cultures [196,238], but higher MICs were observed in the Cip-resistant planktonic cultures [196]. In agreement with the planktonic-only results, biofilm bacteria evolved in the presence of Cip accumulated mutations in type IV pili, leading to impaired twitching motility [164,165,166], while biofilm bacteria evolved in the absence of antibiotics were more likely than their Cip-evolved counterparts to lose QS ability [194,196].

#### 6.2.2. *In Vivo* Studies

##### Co-Operation between *P. aeruginosa* Strains

In humans, Köhler et al. (2010) performed a double-blind randomised controlled trial in which intubated patients newly colonised with *P. aeruginosa* were intravenously administered with either 300 mg/day of AZM or placebo for between 3 and 20 days (average treatment time: 10 days) [168]. AZM was found to significantly reduce QS gene (*lasI* and *rhlA*) expression, as measured directly in tracheal aspirates each day (*p* = 0.006 and *p* = 0.005, respectively). Interestingly, the proportion of *lasR* QS-null mutants significantly increased over time in the patients who were administered placebo (*p* < 0.001), but not in those who were administered AZM. Consistent with these findings, isolates from placebo-treated patients showed decreasing mean elastase levels *in vitro* (*p* < 0.001), while isolates from the AZM-treated group showed an increase over time (*p* < 0.001) [168]. These findings, whereby AZM was administered *in vivo* for an average of 10 days to patients newly colonised with *P. aeruginosa*, support the shorter-term *in vitro* findings that AZM selects against the development of *lasR* mutants, likely by inhibiting QS in wild types [168].

The authors also found from the trial data that QS-dependent virulence was important for the development of infection (as determined by the acquisition of ventilator-associated pneumonia (VAP)). The development of VAP from initial colonization occurred significantly earlier in patients colonised solely by wild types than in those who also harboured *lasR* mutants (*p* = 0.001) [239].

##### Acute Infections

Several animal studies describe the protective effects of sub-MIC exposure to tetracycline, ciprofloxacin, tobramycin, and ceftazidime against *P. aeruginosa* for lung infections in rats and to AZM against *P. aeruginosa* for renal infections in mice and rats [173,186,240]. However, the mechanism by which the antibiotics achieve this protection is not always understood.

##### Chronic Infections

The therapeutic benefits of the long-term administration of various macrolides in diffuse panbronchiolitis patients have been demonstrated in several Japanese studies [241]. For example, erythromycin has been shown to improve respiratory function [242] and chest X-ray pictures [243], relieve patients from productive coughs and dyspnea [243], and increase survival rates [244], with at least part of the mechanism of action thought to be anti-inflammatory [242]. Erythromycin has also proven effective in patients with non-CF bronchiectasis. A 48-week double-blind randomised controlled trial found that erythromycin significantly reduced total pulmonary exacerbations without changing the bacterial load. Part of the mechanism of action may be the inhibition of QS, given that a significant reduction in the expression of the QS genes *lasR* and *pqsA* was witnessed [245].

For CF patients, AZM, Cip, and chloramphenicol are administered orally. Several clinical trials investigating the long-term administration (3–6 months) of AZM have found it to be effective, with benefits including reduced rates of infective exacerbations and a reduced need for courses of intravenous antibiotics [246,247]. Further, a study in intubated (non-CF) patients infected with *P. aeruginosa*—a patient group that is prone to experiencing VAP—found that lower incidences of VAP occurred in AZM-treated than in untreated patients [248].

The mechanism by which AZM exerts its beneficial effects is likely to be multifaceted. AZM has been shown to reduce virulence factor production *in vitro* (Table 1), and this virulence reduction hypothesis is supported by the results from several *in vivo* studies [172,249,250]. One such study found that, *in vivo*, the reduction in bacterial phospholipase C (PLC) production at constant bacterial load was found to significantly correlate with improvements in the lung function measurement, FEV1, in CF patients [172]. The drug may also exert a bacteriostatic effect. Though the findings did not reach statistical significance, the results from a trial in CF patients showed a net reduction in *P. aeruginosa* bacterial density of 0.5 log CFU in AZM-treated patients at day 168 versus placebo [246]. Another way in which the drug may exert its effect is through an anti-inflammatory mechanism, as revealed by studies with CF patients without *P. aeruginosa* infection [251].

Although oral Cip is also frequently given for two-week periods during exacerbations, there is mixed evidence from clinical trial data to suggest that it is effective [252]. In a randomised double-blind placebo-controlled trial, in which 31 patients received Cip or placebo for 10 days every 3 months for 1 year, Cip improved the peak expiratory flow (PEF) versus placebo (*p* = <0.05), but it did not reduce the rate of hospital admissions with acute exacerbations of respiratory symptoms, reduce the time spent off work, or result in improvements in body weight [252]. While improvements in mean FEV1 (the volume of air exhaled at the end of the first second of forced exhalation) and mean FVC (the total amount of air exhaled during the FEV test) were not statistically significant (*p* = >0.05), due to sample size and initial intra-patient variability, significant improvements were shown for self-reported symptoms, including breathlessness, sputum volume, and mean PEF in the Cip-treated group versus placebo (*p* = <0.005) [252]. There is a lack of clinical trial data for the use of chloramphenicol in this patient group, but, again, there is anecdotal evidence of its benefits [253]. For intravenously administered antibiotics, there is limited clinical trial data on their effectiveness, but clinical experience supports their use [253]. Ceftazidime and tobramycin are commonly used together, with meropenem and colistin used as a suitable alternative combination [253].

## 7. The Translatability of *In Vitro* Virulence Assay Findings

Although *in vitro* virulence assays based on sub-MIC antibiotic-treated *P. aeruginosa* cultured in isolation tell us something about the effect of the drugs on virulence factor production, the results are not necessarily translatable to the pathogen’s *in vivo* virulence. Suggestions of this can be seen from studies in which animal tissue has been infected with *P. aeruginosa*. In a series of early experiments by Geers and Baker, hamster trachea organ cultures were used to investigate the ability of the sub-MICs of various antibiotics, including the beta-lactams ceftazidime and carbenicillin, to prevent *P. aeruginosa* infection from damaging the epithelium [121,127]. Although some of the sub-MIC antibiotics prevented tissue damage, the beta-lactams were not able to do so, and, indeed, oftentimes damage still occurred when the antibiotics were administered at or above the MIC. These results are not what we might have expected based on the findings from *in vitro* studies, where the supernatant from *P. aeruginosa* cultured in isolation with these antibiotics was used to test for the presence of various virulence factors. Such studies found the antibiotics to inhibit a host of virulence factors, including exotoxin A (ceftazidime and carbenicillin [122,143]) and elastase (ceftazidime only [143,157,158]), two important virulence factors for inducing host tissue damage [193,194]. However, the testing of the culture supernatants of the *P. aeruginosa*-infected, antibiotic-treated hamster tracheal explants for the presence of exotoxin A and elastase revealed that neither beta-lactam, even at concentrations at or above the MIC, had been able to inhibit their production, which likely goes some way towards explaining the drugs’ inability to prevent *P. aeruginosa* from damaging the infected epithelium [121,127]. Given the role of surface attachment in inducing virulence, the fact that *in vivo P. aeruginosa* will encounter host cells, and that some antibiotics (e.g., macrolides) have been shown to impact host—as well as bacterial—cells, these studies highlight that there is a limit to how much information can be gleaned from solely looking at virulence factor production in *P. aeruginosa* in isolation. A more complete understanding of the bacteria’s virulence likely requires studies to be conducted in a mammalian tissue setting.

That said, there is not necessarily a disconnect between “theoretical” virulence, as measured by biomarkers, and actual host cell-directed virulence. In agreement with findings from longer-term studies of planktonically grown *P. aeruginosa* evolved in sub-MIC Cip where QS was maintained [192], we found that the virulence of *P. aeruginosa* towards HeLa cells (during overnight co-incubation) was maintained throughout a 25-day evolution period when the bacteria were evolved in sub-MIC Cip, but reduced when evolved without it (Behrends and Nolan, *in prep.*). On the resistance end, Oliveira et al. identified a number of triggers of biofilm formation *in vitro* that likely have relevance to clinical settings [220].

Several papers have demonstrated the *in vivo* adaptation of *Pseudomonas*, most prominently to the CF lung [70,71,73], and although there has been significant media development for *in vitro* models of CF lung infection [254], there is a relative paucity of *in vitro* models for urinary tract infection or burn wounds. However, a promising model for the latter has recently been proposed [255], which will provide important insight, particularly if we take into account the polymicrobial nature and resulting host–microbial and microbial–microbial interactions of most infections [20].

## 8. Future Directions

To date, most studies investigating the interactions of pseudomonal virulence factors and sub-MIC antibiotic exposure have been undertaken over the short term, using a single strain in a planktonic state. Although these have led to some mechanistic, clinically relevant insights, the results may not be translatable because we know that, in infection scenarios, the situation is far more complex. For example, the bacteria exhibit a variety of modes of growth, exist as a variety of strains, encounter different environmental niches (such as differing oxygen tension, pH levels, nutrient availability, and antibiotic concentrations), are under attack from the host’s immune system and from competing microorganisms, and may survive over the longer term, establishing chronic infections. Although studies have been undertaken that consider some of these factors, they are limited in number and scope. For example, useful insights have been gained from studies that have been undertaken on surface-attached or biofilm-dwelling bacteria, but there are very few of them. The same can be said of inter-bacterial competition and inter-strain competition/co-operation studies. Of the longer-term (i.e., evolutionary) *in vitro* studies undertaken to date, the focus has largely been on ciprofloxacin, despite many other antibiotics being administered to patients with chronic pseudomonal infections (particularly CF patients). Trial data is also lacking to support the use of many of these antibiotics, and no animal models exist that reliably represent CF lung infections. To advance the field, future studies must therefore consider all of the factors that impact virulence, as well as resistance, and the translatability of the obtained results to a real biological context. In addition to those factors already highlighted, we discuss below some of the other considerations we deem important for future sub-MIC antibiotic exposure studies.

### 8.1. What Is a Sub-MIC?

This question is both central and surprisingly difficult to answer. The MIC itself varies with bacterial strain, testing method, and culture conditions [139,256,257,258]. For example, for AZM, no published breakpoints exist and wide variations in the MIC are reported in the literature, ranging from 0.5 mg/L [165] to 800 mg/L [158], with it frequently found to be greater than or equal to 256 mg/L [124,158,164,259]. However, more recent studies have demonstrated that the MIC of AZM may be several dilutions lower when determined in a culture medium more closely resembling *in vivo* conditions than in traditionally used media. For example, the MIC for wild type PAO1 was found to be between 0.25 and 16 mg/L when the medium normally used for culturing eukaryotic cells was used [228,258,259]. Similarly, low MICs of 1–8 mg/L were exhibited when at least 50% serum was added to the conventional microbiological media of cation-adjusted Muller–Hinton broth (CA-MHB), with the MIC reducing in inverse proportion to the percentage of serum in the medium [258,259]. In comparison, MICs of >64–256 mg/L were determined in CA-MHB alone [228,258,259]. Furthermore, various multidrug-resistant strains of *P. aeruginosa* were found to have MICs between <0.06 and 1 mg/L in RPMI + 5% LB (media used for culturing eukaryotic cells), which were between five and nine dilutions lower than the MICs determined in CA-MHB [228]. It appears the reason for the lower MICs observed in these media is likely a combination of the increased permeability of the outer cell membrane and reduced efflux, leading to a higher accumulation of the antibiotic within the cell [259].

Media composition, therefore, has a major impact on antibiotic efficacy, but deciding which medium to use is not straightforward. Media are often designed to mimic or reflect *in vivo* conditions, but it is important to remember that these are still media and therefore can only ever approximate real-life growth conditions. For the CF lung, two media often used in *in vitro* studies, in an attempt to more closely resemble the *in vivo* situation, are synthetic cystic fibrosis sputum medium (SCFM) [260] and artificial sputum medium (ASM) [261]. Using the former, which was created based on the gas chromatography-mass spectrometry measurement of the small-molecule complement present in the airways of twelve CF patients, the authors were able to reliably replicate several read-outs such as gene expression or QS levels obtained during growth on ex vivo CF sputum [260]. Use of the latter, which was also created based partially on an analysis of sputum amino acids, was shown to recreate CF lung-specific findings, such as phenotypic diversification [84,261]. While SCFM only contains salts and small molecules such as amino acids or lactate, ASM additionally contains pig mucin and DNA, and the two media look nothing alike. Both media have since undergone further developmental iterations and *P. aeruginosa* exhibits distinct differences in virulence factor production and resistance when cultured in each [254,262].

Interestingly, the use of mammalian tissue yields very different results again. During early *in vitro* studies conducted by Baker and Geers, the authors found that the MIC of the aminoglycosides tobramycin and gentamicin and of the beta-lactams carbenicillin and ceftazidime was respectively between two and eight times and 10 and 40 times higher in hamster tracheal explants than in standard broth suspension. This suggests that the bacteria may become more resistant to antibiotics once they have attached to a biotic surface. Even more intriguingly, a synergistic relationship was found to exist between the aminoglycosides and the beta-lactams when the antibiotics were administered to *P. aeruginosa* infecting tracheal explants, which was not apparent when they were administered to *P. aeruginosa* cultured in standard broth suspension [263]. In the organ cultures, the MICs of both tobramycin and ceftazidime were found to be eight times lower when the antibiotics were administered together than when administered in isolation (1 µg/mL and 4 µg/mL, respectively, versus 8 µg/mL and 32 µg/mL) [263]. The same was true of tobramycin in combination with another beta-lactam, carbenicillin (MICs of 1 µg/mL and 40 µg/mL, respectively, when administered together, versus MICs of 8 µg/mL and 320 µg/mL, respectively, when administered in isolation) [263]. Furthermore, the tobramycin (1 µg/mL)/carbenicillin (40 µg/mL) combination was able to provide the complete protection of the tracheal epithelium from destruction by *P. aeruginosa* [263]. Similar results were found for gentamicin in combination with each of the beta-lactams, with the only difference being that the MIC of gentamicin was reduced by half (from 8 µg/mL to 4 µg/mL) when combined with ceftazidime, rather than the larger reduction (of 7/8 ths) witnessed for tobramycin.

For biofilm studies, the interaction between antibiotics and environmental factors has also been shown to be important and needs to be accounted for. The ability of *P. aeruginosa* to form a biofilm has been studied in the presence of the sub-MICs of streptomycin (0.5, 1 and 2 µg/mL) in a variety of environments, including three different culture media (TSB, MHB and LB), different pH levels (acidic (5.9), broadly neutral (7.2), and alkaline (7.9)) and different temperatures (25, 30, and 35 °C) [114,139]. The findings illustrate the context-dependency of antibiotic efficacy. Regardless of the temperature and pH level, sub-MIC streptomycin was unable to inhibit biofilm formation when MHB media was used. Furthermore, when the pH was acidic (pH 5.9), biofilm formation was induced at all temperatures and in all culture media. On the other hand, when the pH was alkaline (pH 7.9), a reduction in biofilm formation was observed in both LB and TSB media when the temperature was 35 °C, with the anti-biofilm effect being slightly higher in TSB than in LB media [114,139].

### 8.2. Can We Weaponise Environmental Composition?

Several studies have pointed to the centrality and plasticity of the TCA cycle for both antibiotic tolerance and virulence. The upregulation of flux through the GS seen *in vivo* [62,203] will impact cellular redox balance and lower NADH levels, as the NADH-generating isocitrate-dehydrogenase and alpha-ketoglutarate dehydrogenase reactions are circumvented. Regarding virulence, GS activity and lowered NADH levels have been linked to the upregulation of the T3SS [206,207]. Is *Pseudomonas* therefore more virulent at the site of infection than we thought, due to its diet at the time?

Furthermore, is physiology a key to bacterial killing? Antibiotics are likely to increase oxidative stress [16]. However, bacteria with activated GS pathways decrease flux into complex I of the ETC, lowering free oxygen radical production and reducing the potential for oxidative stress generation due to oxidative phosphorylation. Interestingly, exposure to sub-MIC Cip leads to the down-regulation of NAD-dependent *icd*-encoded isocitrate dehydrogenase and the up-regulation of NADP-dependent *idh*-encoded isocitrate dehydrogenase [195]. This increases the amount of NAPDH, which is important for oxidative stress defence [264], thereby potentially increasing antibiotic tolerance. For complex II of the ETC, Ahmed et al. (2018) found that Cip exposure leads to increased mutations in *sdhA*, the gene encoding succinate dehydrogenase subunit A, the flavoprotein of complex II [196], which would lead to a broken TCA cycle.

A logical next step, informed by the observed differences in antibiotic efficacy and virulence in different media, together with recent knowledge on how metabolic flux impacts virulence/antibiotic tolerance, is to research the possibility of adjuvants for antibiotics to increase bacterial susceptibility—either via pathway activation or inhibition. *In vitro* and in a mouse model, Peng et al. (2015) demonstrated that supplying alanine and glucose led to the activation of the TCA cycle, changes in redox balance and proton motive force, and, ultimately, to increased uptake of and susceptibility to kanamycin in a range of bacteria [112]. Future research should aim to spatially resolve metabolic interactions and dependencies in biofilms and in infected tissue.

### 8.3. How Do We Avoid Falling Prey to the Garbage in, Garbage out Principle?

Over the last couple of years, technological advances have made it possible to generate data on a scale that is several orders of magnitude higher than even a decade ago. Whether it is genome sequencing, interrogating metabolite concentrations, investigating metabolic fluxes, or quantifying proteomic or transcriptomic changes, the technology exists, and many of these sophisticated analyses are available as an off-the-shelf service.

More data, of course, does not necessarily equate to better data. If the experimental model used to generate the sample does not reflect the aspect of the bacterial infection to be studied, the meaningfulness of the resulting data set will be limited. While this goes back to the earlier point mentioned above about the translatability of findings, it also emphasises a point about multidisciplinary working. As the field moves into the next phase, major advances will come from collaborative projects undertaken with experts from several disciplines, such as proteomics/metabolomics, theoretical biochemistry, mathematics, statistics, and/or physics. Though widely used, -omics data sets are difficult to analyse and sample preparation for bacterial metabolomics measurements is non-trivial [265,266]. Following the findings from their social cheat experiments, Vasse et al. (2017) built a mathematical model to try to quantify the effects of antibiotics on the relative proportions of producers and non-producers in mixed populations. The model was constructed such that the fitness of each subpopulation depended on the cost of production of the public good, its beneficial effects, the population density, and the effects of the antibiotic on bacterial growth [126]. The results revealed that, dependent on the relative sizes of the antagonism effects and the cost of public goods production, the relative fitness of non-producers can vary with the level of antagonism in four qualitatively different ways: monotonically increasing, monotonically decreasing, peaked, or valley shaped [126]. This highlights the sensitivity of the ecosystem (in terms of the balance between producer and non-producer populations) to the antibiotic concentration employed and highlights the dangers of trying to generalise the findings from any one *in vitro* study using a single sub-MIC dose. Vasse et al. thus highlight that a better understanding of ecological stressors, such as sub-MIC antibiotics, on the impact of social evolution in *P. aeruginosa* infection scenarios—and therefore on host-directed virulence—may really only start to be gained from undertaking this type of mathematical modelling, in combination with more sophisticated *in vitro* experiments [126].

## 9. Concluding Statement

Clinicians treating opportunistic pathogens in general—and *P. aeruginosa* in particular—face increasing difficulties, as these pathogens are becoming more resistant and novel antibiotics are in short/no supply. On the research side, we understand the regulation of some virulence factors, even down to the level of the amino acid involved in the conformational change of a regulating kinase. However, we cannot necessarily predict whether that virulence factor will do any damage *in vivo*. Future *in vitro* and animal model studies will therefore need to be sufficiently representative of real-world infection scenarios, so as to be translatable *in vivo*. Recent gains in the understanding of the links between physiology, virulence, and resistance, general advances in computational methods, and cross-disciplinary collaborations offer a great opportunity to advance the field in this direction.
